# Sleep Patterns and Human Brain Health

**DOI:** 10.1177/10738584241309850

**Published:** 2025-01-30

**Authors:** Anders M. Fjell, Kristine B. Walhovd

**Affiliations:** 1 Center for Lifespan Changes in Brain and Cognition, University of Oslo, Oslo, Norway; 2 Computational Radiology and Artificial Intelligence, Department of Radiology and Nuclear Medicine, Oslo University Hospital, Oslo, Norway

**Keywords:** sleep, brain health, cognition, biomarkers, Alzheimer disease, normal aging

## Abstract

It is a widely held opinion that sleep is important for human brain health. Here we examine the evidence for this view, focusing on normal variations in sleep patterns. We discuss the functions of sleep and highlight the paradoxical implications of theories seeing sleep as an adaptive capacity versus the theory that sleep benefits clearance of metabolic waste from the brain. We also evaluate the proposition that sleep plays an active role in consolidation of memories. Finally, we review research on possible effects of chronic sleep deprivation on brain health. We find that the evidence for a causal role of sleep in human brain health is surprisingly weak relative to the amount of attention to sleep in science and society. While there are well-established associations between sleep parameters and aspects of brain health, results are generally not consistent across studies and measures, and it is not clear to what extent alterations in sleep patterns represent symptoms or causes. Especially, the proposition that long sleep (>8 hours) in general is beneficial for long-term brain health in humans seems to lack empirical support. We suggest directions for future research to establish a solid foundation of knowledge about a role of sleep in brain health based on longitudinal studies with frequent sampling, attention to individual differences, and more ecologically valid intervention studies.

## Introduction: Perceptions of the Role of Sleep in Brain Health

**Figure 1.**
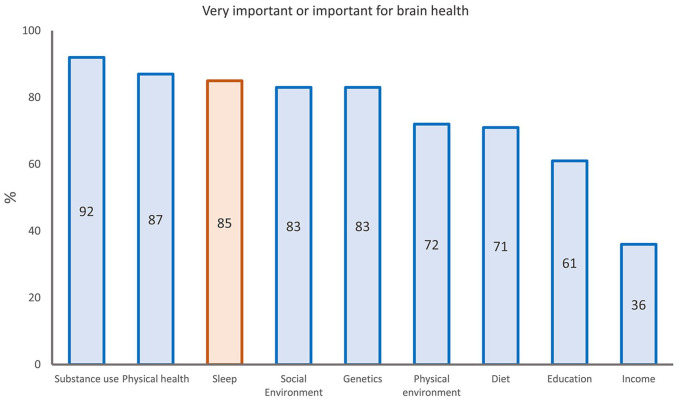
Public opinions about factors important for brain health. The bars show the percentages of 27,500 participants who rated selected factors as “important” or “very important” for brain health, among a broader set of 11 factors. Sleep is highlighted in orange. From Budin-Ljosne and others (2022).

There is a prevailing public preoccupation with the importance of sleep to better brain health. In a survey of 27,500 participants across Europe conducted by the pan-European Lifebrain consortium (Walhovd and others 2018), 85% rated sleep habits as having a “strong” or “very strong” influence on brain health (Budin-Ljosne and others 2022). This number far surpassed the numbers for diet (71%), physical environment (72%), and education (61%; Figure 1), which are all established risk factors for dementia (Livingston and others 2024). Many scientists agree: it has been suggested that treating sleep disorders could delay onset of cognitive decline by a decade (Mander and others 2016) and that 15% of Alzheimer disease (AD) cases may be attributed to sleep problems (Bubu and others 2017). One theory is that sleep facilitates metabolism of amyloid β (Aβ) proteins, the major neuropathologic change in AD, and that sleep problems therefore cause or augment development of AD pathology (Mander and others 2017). The consensus report from the Think Tank World Sleep Forum aligns with this conclusion: “It is well known that neurodegenerative disease cause sleep and circadian abnormalities, that in turn exacerbate and accelerate neurodegeneration” (Ferini-Strambi and others 2024).

**Figure 2.**
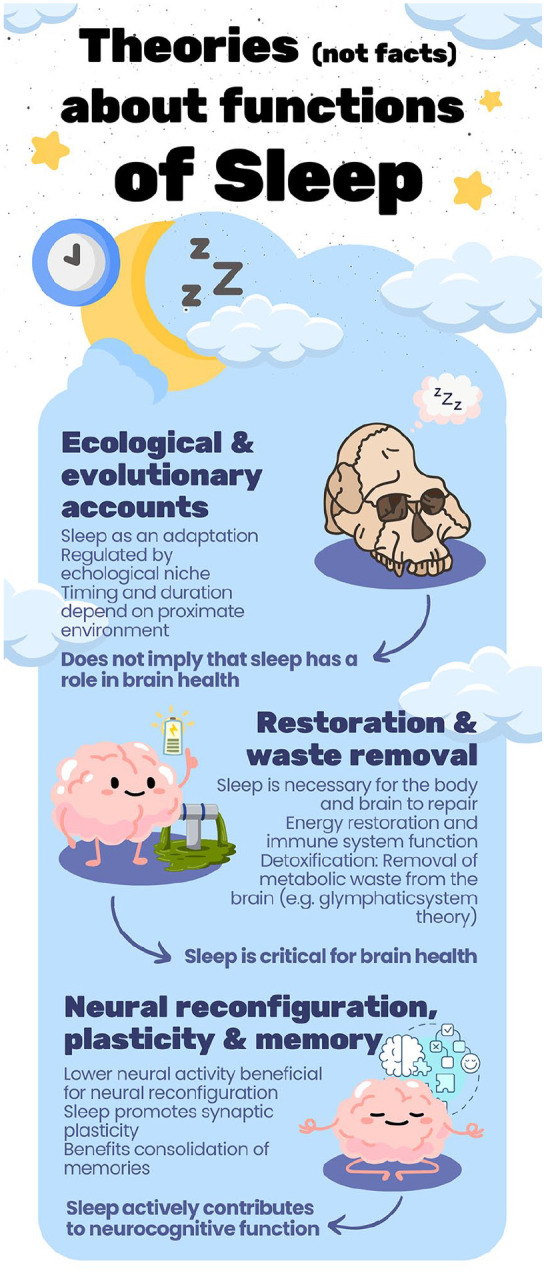
Theories about functions of sleep. There is no agreement about the main functions of sleep or whether sleep serves one fundamental function at all, and many theories exist. For simplicity, we here group them into three categories: ecological and evolutionary accounts; restoration and waste removal theories; and a role in neural reconfiguration, plasticity, and memory. Each group of theories has certain implications regarding the role of sleep in brain health—from the assumed critical role played by sleep according to restoration and waste removal theories to the ecological and evolutionary accounts, which do not necessarily assume that sleep needs to play any role in brain health. All the theories are highly debated. We thank Inge K. Amlien for making the figure.

In this review, we examine the evidence for this widely held opinion of sleep as being critical to good brain health. Brain health can be defined to include most mental functions and neurologic disorders. We focus on more permanent or slower brain and cognitive changes and do not discuss short-term transitory effects of acute sleep deprivation on sleepiness, synaptic plasticity, attentional glimpses, or other cognitive state mechanisms or possible effects of sleep on emotion regulation and well-being. We focus on normal variations in sleep patterns, not sleep diseases and disorders such as obstructive sleep apnea. To evaluate the relationship between sleep and brain health, we need to discuss theories about the fundamental functions of sleep. As we outline here, these theories may have paradoxical implications for the role of sleep in human brain health. For an overview of theories about the functions of sleep, see Box 1 and Figure 2. For simplicity, we divide the theories in three broad categories: 1) ecological and evolutionary theories; 2) theories related to restoration and removal of metabolic waste products from the brain; and 3) theories related to neural reconfiguration, such as synaptic plasticity, memory consolidation, and emotion regulation. Although the last arguably sees effects of sleep on the brain as more transitory, the amount of focus on the association between sleep and memory warrants a short discussion of current opinions about this question. Finally, we review and discuss possible effects of chronic sleep deprivation on human brain health.

**Box 1.** Functions of Sleep.

Several theories about why we sleep exist, and it is even argued that sleep is the only known universal behavior across animals, lacking consensus regarding its fundamental underlying function (Schmidt 2014). For simplicity, here we group three theories about the functions of sleep (for a more comprehensive discussion, see Bodizs 2021). *Ecological and evolutionary theories.* These theories see sleep as an important adaptation, which is regulated by a species’s ecological niche. Hence, sleep varies across species as a function of the specific environmental and survival needs of an organism. For example, sleep patterns may be regulated by predation risk and availability of food. Circadian rhythms are also seen as being shaped in interaction with the environment so that timing and duration of sleep are regulated so ensure that sleep is happening when benefits of being awake are the lowest. For humans, sleep may be most advantageous at night, when visibility is poor. Hence, large variations in sleep between and within species can be explained by the demands of the proximate environment and do not necessarily reflect the brain’s varying need for rest. There is enormous variation in sleep duration among mammals, spanning 2 hours (African elephants) to 20 hours (bats, thick-tailed opossum) per 24-hour cycle, which is not related to brain size or cognitive ability. For example, African elephants and bottlenose dolphins have different and shorter sleep than humans while having larger brains. At the same time, humans sleep the least of all primates, with considerable larger brain volumes and more neurons. Equally important, there is large variation in sleep duration within the same species, with different amounts of sleep across seasons and in response to different environments. For example, animals sleep much more in captivity than in the wild, without any obvious benefits for cognition, alertness, or health. Ecological and evolutionary theories can therefore account for important aspects of animal and human sleep patterns. An overview of contemporary ecological and evolutionary theories of sleep is presented by Siegel (2022). Additional useful contributions include those by Capellini and others (2008), Siegel (2008), Horne (2011), Samson and Nunn (2015), Rattenborg and others (2017), and Samson (2021). Implications of these theories for human cognition are also discussed by Fjell and Walhovd (2024). For a different perspective, see Walker (2021). *Restoration and waste removal theories.* There are multiple physiologic changes during sleep, including body temperature, metabolism, and heart rate. This set of theories sees sleep as being necessary for the body and brain to repair, recover, and restore energy and function after periods of wakefulness. Specific suggestions include that sleep benefits tissue repair, immune system function, energy conservation through lower metabolism, and physical recovery. For the brain, the strongest focus has been on removal of metabolic waste products accumulating during awake states due to higher neural activity. The brain is characterized by very different activity levels across the awake and different sleep stages, accompanied by differences in flow of blood and CSF and increases in perivascular and extracellular space, interstitial in particular. According to restoration and waste removal theories, these sleep-dependent changes in the brain may yield an optimal environment for “detoxification” of the brain necessary after awaking periods. In contrast to ecological theories, these theories more directly connect sleep to brain health. While not always mutually exclusive, the different focus of ecological and waste removal theories can lead to paradoxical implications for human brain health, as discussed in detail in the text. *Theories of neural reconfiguration, plasticity, and memory.* The lower neural activity during sleep may create a time window beneficial for neural configuration, enhanced plasticity, and strengthening of memories. In contrast to restoration theories, it is suggested that sleep actively contributes to cognitive processes. This can include downscaling synaptic strength and pruning of less important synapses during slow-wave sleep, where neural activity is lower. Relatedly, sleep is thought to facilitate synaptic plasticity by promoting long-term potentiation, a fundamental mechanism underlying learning on the molecular level. A particularly popular version of these theories holds that sleep facilitates the transfer of memories from short-term storage in the hippocampus to long-term storage in the cortex. An assumed mechanism extensively researched in rodents is replay of activity patterns during sleep that were experienced during wakefulness. This is thought to strengthen pathways associated with newly learned information, thereby consolidating new memories. To what extent sleep benefits memory consolidation in humans is discussed in the text.

## Sleep as Adaptive and Balancing Individual Needs to the External Environment

According to the ecological and evolutionary theories, sleep is tightly regulated by the ecological niche (Capellini and others 2008). Sleep is seen as adaptive, and functions of sleep are related to external factors, such as food availability (Siegel 2022) and predation risk (Samson 2021). It is noted that sleep may be surprisingly well preserved across species and that sleep-awake regulation is likely not fundamentally different in humans as compared with most other animals (Bodisz 2021). The universality of sleep has been questioned (Siegel 2008), but the prevalence of sleep across species is taken by others to mean that sleep may have one primary function, which is preserved through evolution (Schmidt 2014). If this is true, it is unlikely that humans primarily sleep to promote the health of their large and highly complex brain, as common neural features of sleep exist from insects to mammals, such as silencing, oscillations, and wake-like activity (Jaggard and others 2021). Even animals lacking a centralized brain show sleep-like patterns, which suggests that sleeping predates cephalization (Jaggard and others 2021). Also, the length of sleeplessness in some highly cephalized animals, such as killer whales during development and migration, suggests that the complexity of a central nervous system itself does not dictate a set amount of regularized sleep. Thus, some ecological and evolutionary theories may be incompatible with theories of restoration as the main function of sleep.

An alternative view is that sleep is best seen as a multilevel functional system and that functions of sleep multiplied during evolution (Bodizs 2021). Accordingly, sleep may serve several functions, and even if sleep emerged serving one primary function, it could have evolved into a complex and multidimensional state essential for many aspects of health through opportunistic evolution. These could include specific restoritative processes in the human brain. This view is supported by studies across species demonstrating that although common neural features exist, sleep patterns, behavior, architecture, and functions are not uniform but rather vary widely, which may reflect that they have evolved to meet the specific needs of different organisms (Capellini and others 2008).

In this discussion, it is critical to consider that sleep is not one thing but consists of repeated cycles of stages characterized by different types and levels of brain activity. Theories about functions of sleep need to be able to account for this variation in brain activity, especially the clear division between rapid eye movement (REM)—or paradoxical sleep, where brain activity resembles awake states—and non-REM sleep. Such a division not only characterizes human sleep but is seen across mammals and in birds (Schmidt 2014; Siegel 2022). The conservation of sleep stages during evolution across many, but not all, animals suggests that they serve some specific functions. For example, the sleep intensity theory suggests that characteristics of human sleep emerged as a response to a selection pressure to fulfill sleep needs in the shortest time possible, due to predation risk associated with sleeping in terrestrial environments, threats from conflicts, and increased benefits of being awake with the emergence of more social interaction and transfer of knowledge and skills (Samson and Nunn 2015). The theory explicitly addresses sleep architecture and proposes that human sleep is shorter, more intense, and with a higher proportion of REM as compared with other primates. This is compatible with a view of sleep as being necessary for brain restoration.

In contrast, it has been argued that sleep primarily reflects ecological constraints, which act on total sleep duration rather than selectively affecting different aspects of sleep resulting from specific selection pressures (Capellini and others 2008). Accordingly, variations in sleep would likely have minimal impact on brain health. A third suggestion is that the function of REM sleep is temperature regulation of the brain and preparation for wakefulness in endotherms (Siegel 2022), not otherwise being important for brain health. This is supported by experimental studies of animals.

Ecological theories of sleep are powerful in explaining the wide diversity in sleep behavior and architecture across species and societies, but they often do not offer specific predictions about a relationship between sleep and brain health, except for highlighting the possibility that there may not be any. Interestingly, it is sometimes argued that sleep must serve a critical function for bodily and brain health, precisely because sleep otherwise would be “the biggest mistake the evolutionary process has ever made” (Walker 2021). However, as we briefly summarize so far, many ecological and evolutionary theories ascribe a critical evolutionary role to the emergence of sleep without making assumptions of unique contributions of sleep to health (see Siegel 2022 for an overview). Still, even if the ultimate function of sleep evolved in response to the ecological niche of earlier humans, the possibility of sleep affecting brain health cannot a priori be excluded. We continue with a discussion of the theory of sleep as being important for restoration of the brain.

## Sleep as Regulating Metabolic Waste in the Brain

### Is There Increased Metabolic Waste Removal from the Brain During Sleep?

Restoration theories assume that sleep plays a crucial role for brain health, typically by facilitating removal of metabolic waste products from the brain, which accumulates during awake states. The fundamental idea is >100 years old (Bodizs 2021), and since the 1970s, more specific hypotheses have been put forth about a “waste removal” system (Sangalli and Boggero 2023). This is highly relevant, as many neurodegenerative conditions are associated with disrupted sleep and changes in sleep patterns are sometimes observed years before cognitive decline (Wang and others 2024). Waste clearance theories suggest that sleep disturbance itself may play a causal role in neurodegeneration, typically by focusing on interactions between interstitial and cerebrospinal fluid during sleep. The current leading waste clearance theory is the theory about the “glymphatic system.”

#### A Glymphatic System for Waste Removal?

**Figure 3.**
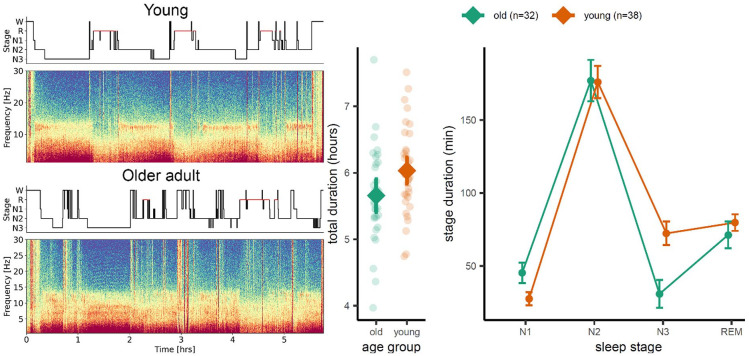
Age differences in sleep architecture. Older adults show on average less deep sleep (stage N3, slow-wave sleep) than younger ones. The left panel shows a spectrogram for a young (top) and older (bottom) healthy adult. The scatter plot in the middle panel shows that there was no significant difference in total sleep duration between groups of 32 older and 38 younger adults but that the older adults still spent significantly shorter time in N3 sleep than their younger counterparts (right panel). Time spent in other sleep stages did not differ. Unpublished results from the Center for Lifespan Changes in Brain and Cognition (courtesy of Liisa Raud).

Waste removal theories gained increased popularity with the proposition of the glymphatic system (Iliff and others 2012). It was suggested that sleep drives metabolic clearance from the mouse brain by an increase in convective exhange of cerebrospinal fluid with interstitial fluid (Xie and others 2013). Waste products, including Aβ, tau, and α-synuclein, are generated by synaptic activity during active wakefulness, causing the concentration of these metabolites to increase in the extracellural fluid and CSF during wakefulness (Nedergaard and Goldman 2020). Fluid flow through the glymphatic system is most efficient during deep sleep (Ju and others 2017) and stops completely with the onset of wakefulness (Nedergaard and Goldman 2020). The energetic cost of waste clearance is largely independent of metabolic concentrations, which makes it more efficient to initiate clearance after metabolic waste has been built up (Bodizs 2021). Consequently, it follows from this theory that disturbed deep sleep has a causal effect on the development and progression of neurodegenerative disorders through reduced clearance, leading to accumulation of metabolic waste and downstream effects such as brain atrophy and cognitive decline. Aging is also associated with less deep sleep (Redline and others 2004), as seen in Figure 3, which according to this account will reduce the amount of metabolic waste removed every night. The theory hence provides an explanation for the dramatic increase in dementia prevalence with age.

Although the theory gained popularity, there are caveats. The orginal discovery of the glymphatic system continues to be controversial (Reardon 2024). A review concluded that “the major mechanistic aspects of the glymphatic hypothesis are highly speculative and perhaps incorrect” (Smith and Verkman 2018: 549), suggesting that use of the term “paravascular system” would be more appropriate and that waste clearance more likely depends on nondirectional diffusion than convective active fluid transport (but see, e.g., Kipnis 2024). Additionally, recent studies have argued for reduced, not increased, waste clearance during sleep (Miao and others 2024) and general anesthesia (Gakuba and others 2018). In the present context, it is also important to critically evaluate whether the implications of the glymphatic theory hold up in studies of human brain health, which we discuss next.

#### Does the Glymphatic System Theory Apply to Human Brain Health?

The glymphatic theory focuses on deep sleep (slow-wave sleep [SWS]) and does not account for established associations between brain health and other sleep stages, such as REM or total sleep time. A recent meta-analysis of studies using objective sleep measures in patients with mild cognitive impairment found SWS to be reduced by 0.78% as compared with 2.69% for REM (Wei and others 2024). Why REM sleep is reduced in mild cognitive impairment is not explained by the theory, and it is unclear whether the small reduction in SWS is sufficient to have a major impact on metabolic waste clearance. Along the same lines, another meta-analysis (Zhang and others 2022) and a large community-based study (Pase and others 2017) found greater cognitive decline with REM but not SWS disturbance.

It is unclear how the glymphatic theory accounts for associations between brain health and variations in habitual sleep duration. Brain metabolic activity during REM sleep is on level with awake activity (Maquet 1995), likely yielding similar metabolic waste production. Given that the major share of SWS occurs during the first 4 hours of sleep while REM dominates the last part of the night, longer sleep should not affect metabolic waste removal to any substantial degree. As change in sleep duration predicts development of dementia (Bubu and others 2017; Cavailles and others 2022), there must be more to the relationship between sleep and neurodegeneration than the glymphatic system.

Most important, there is not convincing evidence for a specific association between disturbed SWS in humans and accumulation of AD biomarkers such as Aβ and tau from neither experimental nor observational studies (Kimura and others 2023; Sangalli and Boggero 2023; see Box 2 for a background on CSF biomarkers for AD). Observational studies have the advantage in that they do not interfere with normal sleep and hence have higher ecological validity (Fjell and Walhovd 2024), but the caveat is that they lack experimental control. Such studies often report no or opposing relationships among Aβ, tau, and sleep parameters measured by self-report (Baril and others 2024), actigraphy (Lim and others 2013; but see Blackman and others 2022), or polysomnography (Kimura and others 2023; Sangalli and Boggero 2023). A quasi-experimental study of maritime pilots found no effects on global brain Aβ levels or cognitive function of long-term intermittent sleep disruption (Thomas and others 2020).

**Box 2.** CSF Biomarkers in Alzheimer Disease.

**Figure 4.**
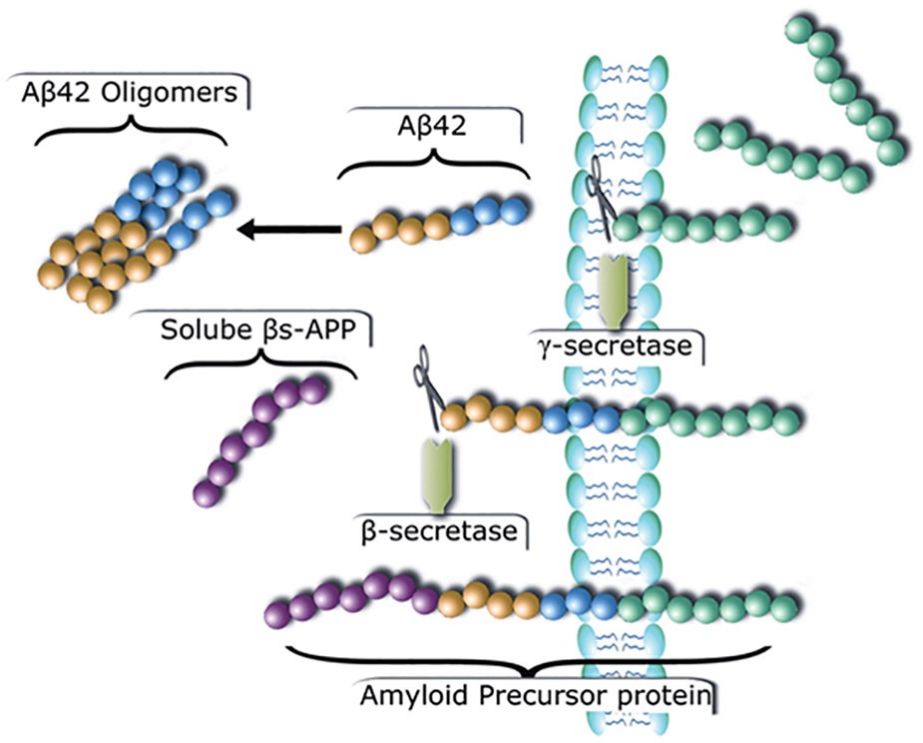
Generation of Aβ42 by cleavage of the amyloid precursor protein (APP). APP is a transmembrane protein and can be cleaved by the γ-secretase pathway, which is nonamyloidogenic, or the β-secretase pathway, which is amyloidogenic. β-secretase cleaves APP before the Aβ domain, and this releases the soluble β-APP (purple circles). The remaining part of the APP (β–C-terminal fragment; β-CTF: yellow, blue, and green circles) is cleaved further by the γ-secretase complex, releasing the free Aβ peptide consisting of 40 to 42 amino acids (yellow and blue circles). The longer isoforms of 42 amino acids (Aβ42) are more hydrophobic and aggregate more rapidly than other Aβ isoforms (e.g., Aβ40). The remaining APP (APP intracellular domain: green circles) is released into the cytoplasm. We thank Inge K. Amlien for making the figure. Printed with permission from Fjell and Walhovd (2011).

Accumulation of Aβ protein in extracellular plaques and hyperphosphorylated tau (p-tau) protein forming intracellular neurofibrillary tangles are the main neuropathologic changes characterizing Alzheimer disease (AD; Goedert and Spillantini 2006). These usually precede accelerated atrophy in the medial temporal lobe, memory decline, and clinical symptoms. Biomarkers reflecting these changes exist, and it is now proposed that biomarker status alone should be sufficient to diagnose and stage AD (Jack and others 2024). Aβ (Aβ42) and p-tau can be measured in CSF, while biomarkers of atrophy can be based on CSF (e.g., total-tau [t-tau], neurofilament light) and serial MRI of the brain. AD biomarkers can increasingly be measured in blood (Barthelemy and others 2024) and Aβ accumulation and tau load by PET. As blood-based AD biomarkers are still not much used in the context of sleep and PET reflects relatively slow brain changes, we focus on CSF markers. Of special interest is CSF Aβ42. As illustrated in Figure 4, Aβ is produced from amyloid precursor protein (APP) by sequential cleavage and is secreted to CSF as a soluble peptide as part of normal APP metabolism (Portelius and others 2008). Depending on where APP is cleaved, Aβ has different isoforms, and the longest (Aβ42) consists of 42 amino acids. Aβ42 aggregates more rapidly than other Aβ isoforms and is the main CSF biomarker of AD. Importantly, the CSF level of Aβ42 is inversely related to the amounts of Aβ42 in the brain. The assumed mechanism is that aggregation of Aβ in plaques reduces the amount of Aβ42 free to diffuse to the CSF; hence, low concentrations of CSF Aβ42 are taken to indicate high brain levels of Aβ42. This fits knowledge that sporadic AD is related to reduced clearance of Aβ, resulting in amyloid plaques. For p-tau, higher CSF concentrations indicate higher brain levels of p-tau. In the context of sleep experiments, the issue is more complicated, as the outcome is dynamic short-time changes in biomarker levels (Box 3). In contrast to neuropathologic examinations and amyloid PET scans, which index plaque load formed over years, CSF levels of Aβ42 reflect the continuous balance of production, clearance, and degradation. This means that short-term changes in concentration can result from alterations in either of these processes or their interactions. Hence, while low levels of CSF Aβ42 normally reflect deficient clearance and high plaque load, the interpretation is less straightforward in the context of short-term experimental manipulations, with unclear implications for brain health. Similar concerns regard other biomarkers than Aβ42. See Box 3 for a discussion of the use of CSF markers in experimental sleep research.

In contrast to observational studies, true experimental studies can adress causality by comparing concentrations of metabolic waste in CSF in conditions where SWS is hindered or not. We include a detailed discussion of some of the most important studies in Box 3 and focus on the main conclusions here. An inherent issue is that higher production, processing, and release of certain metabolies and higher clearance may raise CSF concentrations. Hence, as sleep deprivation is associated with higher neural activity than normal sleep, we would expect higher CSF concentrations of these metabolites after sleep deprivation. This is, for example, consistent with the diurnal pattern of lower CSF Aβ42 levels in the morning (Huang and others 2012) and higher levels with shorter SWS (Varga and others 2016). However, given the assumption of the glymphatic theory that clearance is higher during sleep, the opposite pattern could also be expected: higher CSF metabolite concentration after sleep than awake due to clearance from the brain to the CSF. Although there is a limited number of studies adressing this, evidence so far does not favor this explanation. Interpretation is challenging, however, because Aβ42 production per se and clearance from the CSF to the periphery through the blood-brain barrier can work at short time scales of minutes to hours (Cheng and others 2020), which implies that changes in either or both likely have profound effects on short-term fluctuations in CSF levels.

**Box 3.** Sleep Deprivation Studies of CSF Biomarkers.

A 25%–30% increase in CSF levels of Aβ38, Aβ40, and Aβ42 was found in seven sleep-deprived participants as compared with sleeping controls (Lucey and others 2018), interpreted as reflecting Aβ production, not primarily reduced clearance. An increase in several tau species was observed in the same study, reflecting greater processing and release during sleep deprivation but not higher production (Barthelemy and others 2020). A later study also found 35%–55% greater CSF concentration of Aβ and tau biomarkers (Liu and others 2023). Although the interactions among production, clearance, and CSF kinetics are complex, if sleep yields more efficient clearance, one could according to the glymphatic theory expect lower CSF concentrations in the sleep-deprived group as a result of less Aβ42 cleared from the brain with the lack of slow-wave sleep (SWS). A recent study showed markedly reduced clearance of fluorescent molecules during sleep and anesthesia in mice (Miao and others 2024) in accordance with the aforementioned results. The mechanisms are not clear, but the authors speculated that CSF outflow from the brain could be reduced by anesthetics and that a rapid CSF turnover through lymphatics precluded significant bulk flow to the brain (Ma and others 2019). Different results were seen with a 6% decrease in CSF Aβ42 in 13 sleep-deprived participants versus 13 sleeping controls but with no effects on other Aβ and tau markers (Ooms and others 2014). This could be consistent with reduced AB42 production during sleep. A complicating issue was that there were numeric baseline differences between the groups in protein levels measured in the evening, before the experimental manipulation. Taking these differences into account, we found no significant difference in the change of Aβ42 levels between the sleep group and sleep deprivation group (according to our own reanalyses of the published data), which does not suggest more efficient metabolic clearance during sleep than an extended awake period. A study using partial sleep deprivation (<4 hours per night) for five consecutive nights found a 27% increase in CSF orexin concentrations but observed no changes in CSF biomarkers for amyloid up-building, Alzheimer disease–type neurodegeneration, or astroglial activation (Olsson and others 2018). The authors concluded that there were no major effects of partial sleep deprivation on the turnover of these proteins within the CNS. A caveat was that the partial sleep deprivation led to a shortening of time spent in all sleep stages except SWS, which makes it difficult to draw conclusions regarding impact of the glymphatic system for these biomarkers as waste clearance would be expected to happen during SWS. A different experimental approach was taken in a study where SWS disruption was induced by playing tones to the participants (Ju and others 2017). The results showed an association between SWS disruption and higher CSF Aβ40 but not Aβ42, total protein, tau, YKL-40, or hypocretin, likely caused by changes in neuronal activity during disrupted sleep. As there was an increase in the disruption condition and not a reduction in the sham condition, the results do not fit with the glymphatic theory of waste removal during SWS. This interpretation was supported by no correlation between total sleep time and Aβ40 change. It is unclear why disrupted SWS should lead to more of an increase in Aβ levels than the normal awake state, but repeated sampling per se may possibly affect concentrations (see further mention of this issue below). The authors commented, “That total protein levels in CSF were unaffected suggests there were no global effects of [slow-wave activity] disruption on bulk flow mechanisms by which albumin and other abundant proteins enter and exit the CNS.” It must be noted that results from artificialy induced SWS disruption protocols do not automatically generalize to natural occuring conditions associated with SWS disruptions. Repeated CSF sampling within short time intervals may affect fluid dynamics of the CSF (Lucey 2020), possibly causing a monotonous increase of metabolite concentrations (Huang and others 2012). An observational study using repeated CSF sampling found the strongest circadian amplitude in young participants, with decreased amplitude in older participants who were Aβ negative and even lower amplitude in older individuals who were Aβ positive (Huang and others 2012). A steady increase in Aβ levels was seen, which was ascribed to interrupted sleep, cumulatively greater stress in the participants, or changes in CSF flow pathways as a result of the sample’s rising lumbar Aβ concentrations to a level approximating brain or subarachnoid CSF. It is therefore often difficult to tease apart the effects of sleep and sleep deprivation from naturally occuring circadian and diurnal patterns (but see Orduna Dolado and others 2024).

An influential study found increased CSF concentrations of multiple Aβ species in sleep-deprived participants, including Aβ42 (Lucey and others 2018). The authors argued that this reflected higher Aβ production and concluded that the results “unequivocally show that glymphatic clearance alone, without compensation from other clearance mechanisms, would be ineffective in protecting the brain from AD.” In contrast, they interpreted higher levels of tau to suggest greater processing and release during sleep deprivation but not increased production (Barthelemy and others 2020). Importantly for the present discussion, there was no evidence for more efficient clearance of CSF metabolite concentrations during sleep. Higher Aβ and tau concentrations after sleep deprivation have been seen in other studies (Liu and others 2023) but not unequivocally (see, e.g., Olsson and others 2018; Ooms and others 2014). Furthermore, a study using artificial SWS disruption yielded mixed results, concluding that effects were specific to neuronally derived proteins and hence likely caused by changes in neuronal activity during disrupted sleep (Ju and others 2017).

A methodological complication is that repeated sampling may affect fluid dynamics in the CSF (Lucey 2020), in accordance with the often-observed finding of linear increases in Aβ and tau concentrations with frequent sampling (Huang and others 2012). It is therefore difficult to tease apart effects of sleep and CSF sampling per se from naturally occuring circadian and diurnal patterns. A recent observational study overcame these limitations by counterbalancing morning/evening sampling and separating them by 53 days on average (Orduna Dolado and others 2024). Contrary to what could be expected if clearance were higher during sleep than awake, 17% higher CSF Aβ42 and 10% higher Aβ40 concentrations were seen in evening samples as compared with morning. Several synaptic and endolysosomal proteins also increased in concentration, while others, including tau, were unaffected.

Although the results to date are not completely consistent and are subject to different interpretations, we believe that they in general do not support the glymphatic theory of waste removal during SWS, and there are few indications that sleep deprivation reduces metabolic waste clearance. The mechanism most consistent with the findings in the literature seems to be as follows: 1) awake → more neural activity → more waste production → more clearance → higher concentrations of metabolites in CSF in the evening and after sleep deprivation and 2) sleep → less activity → less production → less clearance → lower levels of metabolites in CSF after sleep. From this account alone, it is not necessary to assign a special role of sleep in waste clearance. However, it is still possible that lower Aβ42 CSF levels after sleep partly reflect efficient removal from CSF through drainage pathways into the blood or lymphatic system. The main mechanisms through which Aβ leaves the brain and the contributions of several pathways to overall Aβ clearance are not yet known (Cheng and others 2020), complicating interpretation of short-term variations in CSF levels of Aβ. There are also possible increases in interstitial space during sleep, which could allow for more efficience clearance from the brain to the CSF, but more evidence is needed before one can conclude that this plays a role in human brain health.

An alternative approach to the use of fluid biomarkers is brain imaging. There is a paucity of PET studies, but one found significantly greater uptake of ^18^F-florbetaben, which binds to fibrillary Aβ, in thalamus and a smaller part of the hippocampus after acute sleep deprivation as compared with normal sleep in 20 healthy participants (Shokri-Kojori and others 2018). These results are interesting, but questions remain: ^18^F-florbetaben binds to insoluble Aβ, and it is not clear what short-term increases in uptake mean. Furthermore, the main part of the voxel-wise effect was seen in the thalamus, which typically does not show Aβ deposits very early in the evolution of β-amyloidosis (Thal and others 2002), nor does the hippocampus, the other effect site in the study. The mean age of the participants was 43 years, where amyloid positivity is very rare, so Aβ changes at this age have unknown implications for aging-related neurodegeneration and later cognitive decline. The authors speculated that the Aβ increases reflected greater Aβ synthesis and production associated with endogenous neuronal activity in the hippocampus during sleep deprivation, in line with evidence from mice suggesting that local Aβ aggregation can be driven by endogenous neuronal activity, which regulates the regional concentration of interstitial fluid Aβ (Bero and others 2011). If this is correct, different clearance rates may not be responsible for the observed group effect. A similar issue applies to a study using MRI to compare concentrations of a contrast agent, gadobutrol, after sleep versus sleep deprivation (Eide and others 2021). In several regions, concentrations were higher after sleep dreprivation than sleep, but it was also clear that full uptake was not achieved at the time of the last scan before the experimenal maniuplation in many brain regions; thus, it is not immediately possible to attribute postintervention differences to differences in rate of clearance alone.

In conclusion, human studies to date do not provide consistent evidence to support a role of sleep in metabolic waste clearance from the brain. The elevated concentration of metabolites in CSF is consistent with increased production during awake and reduced clearance during sleep (Miao and others 2024), opposite of a natural prediction from the glymphatic system theory. Still, interpretation is challenging due to limited knowledge about clearance from the brain and from CSF to the peripery. Rather than through clearance, it is possible that sleep contributes to less metabolic waste because of lower neuronal activity during deep sleep, which we now discuss.

## Is Metabolic Waste Production in the Brain Reduced during Sleep?

An early PET study found that glocuse metabolism was reduced by 43.8% during SWS as compared with wakefulness (Maquet and others 1990). Deep sleep hence can be expected to cause less metabolic waste due to lower neural activity than during wakefulness. Conversely, rodent studies find that sleep deprivation raises levels of proteints that are released with neuronal activity, such as Aβ, tau, and α-synuclein, but not other proteins, such as neurofilament light (Holth and others 2019). By this mechanism, increased wakefulness could eventually raise amyloid deposition via enhanced Aβ production from neuronal activity (Lucey 2020). As mentioned earlier, sleep-mediated changes in tau concentrations are most likely caused by altered release rather than production. Specifically, tau has a half-life of ~23 days in humans after translation, but its half-life is on the order of hours after it is released into the brain insterstitual fluid or CSF (Lucey 2020), as compared with ~9 hours for Aβ.

Although the link between neural activity and waste production is well estalished, there are unanswered questions in the context of sleep and brain health. First, brain metabolism is on level with awake activity during most sleep stages, such as REM sleep, deviating notably only during SWS. Partial sleep deprivation, sleep restriction, and habitual short sleep primarily affect light sleep (N1, N2) and to some extent REM sleep but typically not SWS, limiting the effects on metabolic waste production. Thus, although increased production and other processes could affect levels of metabolic waste in situations where SWS is disturbed, this account is not generally applicable to observed relationships between other aspects of habitual sleep and declining brain health.

Total time spent in SWS is typically between 1 and 2 hours each night (e.g., 96 minutes for young adults in the study by Bes and others 1991), during which time neural activity may be reduced by ~50%. If we assume that metabolic waste production is also reduced by 50%, this yields a reduction of ~3.1% due to total supression of SWS during a 24-hour cycle. If production-release-clearance otherwise is in perfect balance, this could over time lead to substantial accumulation of waste if clearance is not increased with increased need. Still, 3.1% is a much smaller increase than what is typically reported in sleep deprivation studies, suggesting that this cannot be the full explanation for higher biomarker concentrations in such studies.

The neural activity–level account, whether accurate or not, implies that sleep serves no specific function in metabolic waste clearance. This is at odds with the theory of the glymphatic system and the concept of sleep as facilitating waste clearance from the brain. While appealing in its simplicity, it likely does not represent the full story, because without dynamic clearance mechanisms, each night of insufficient sleep could lead to lasting higher levels of metabolic waste.

A further concern is what type of relationships can be expected among sleep, biomarkers, and brain health decline. A recent study found that it takes on average 16.5 years from an abnormal Aβ PET scan finding until Aβ levels are on par with mild/moderate AD (Burnham and others 2024). Given that β-amyloid accumulates slowly into plaques, it is not to be expected that disturbed sleep will have rapid effects on amyloid plaque concentrations in the brain. The role of rapid fluctuations in CSF biomarker levels caused by sleep disturbance in development of dementia and neurodegeneration is hence unclear. Although it is assumed that increased Aβ production may lead to faster formation of Aβ plaques, this has not been demonstrated in humans without specific genetic mutations. Hence, accumulation of AD pathology biomarkers may be too slow to be validly detected by sleep deprivation experiments. Althoug observational studies can detect such general relationships between AD biomarkers and poorer subjective sleep quality, the direction of causality is unclear, and even correlational results are inconsistent (Sprecher and others 2015; Sprecher and others 2017; Winer and others 2019). In contrast, effects of sleep on brain plasticity and memory are assumed to work at a much faster scale and are therefore much easier to adress by sleep experiments. This is discussed next.

## A Role of Sleep in Memory Consolidation?

Theories assuming a role for sleep in neural reconfiguration link homeostatic regulation of sleep to brain plasticity and learning (Born and Feld 2012). For example, global downscaling of synaptic strength during SWS may be necessary to counter synaptic potentiation and associated growth during awake brain activity (Tononi and Cirelli 2003), which otherwise could exceed available resources. The view that sleep is critical for long-term memory dates back at least to the iconic work of Ebbinghaus on forgetting curves from 1885. A century ago, Jenkins and Dallenbach (1924) directly tested the hypothesis that sleeps benefits memory, demonstrating improved memory for a list of nonsense syllables after a period of sleep. It was later proposed that sleep benefits multiple cognitive functions as well as brain plasticity, but the theory that sleep is important for memory consolidation has received a lion’s share of attention. This is a large topic, and we have space to briefly discuss this here.

We do know that sleep between encoding and retrieval tends to facilitate memory and protect against retroactive interference (Paller and others 2021). The active systems consolidation hypothesis predicts that sleep strengthens the contextual binding of memories, which has been shown for source memory for spatial positions, for example (Berres and others 2024). In line with this, functional neuroimaging studies have shown postsleep changes in hippocampal-cortical functional connectivity (Gais and others 2007). However, there is not agreement about whether the behavioral improvements after sleep are due to sleep playing an active role in memory consolidation (Staresina 2024) or whether sleep merely reflects reduced interference (Siegel 2021; Vertes and Siegel 2005; Yonelinas and others 2019), which also could be attained by restful waking (Gottselig and others 2004). If sleep plays an active role in memory, it is necessary to consider which aspects of sleep are responsible for this.

A role of REM sleep in memory consolidation has received substantial attention, but at least for declarative memories, this theory lacks strong support. The positive effect on declarative memory recall often requires only a short nap (Tucker and others 2006), with insufficient time to reach REM sleep. Furthermore, a case study described a man with a localized pontine lesion caused by a gunshot who had almost no REM sleep but preserved memory function (Lavie and others 1984). After his injury, he was able to complete high school, graduate from law school, and practice law, clearly demonstrating that REM sleep is not a prerequisite for learning or consolidation. Later research focused more on the role of REM sleep for nondeclarative memories, and it has been proposed that REM sleep especially facilitates procedural memory (Smith 2001).

Attention has turned more to the role of SWS in memory consolidation, especially for declarative memories. Results are not clear regarding the size or nature of this effect, however. For example, there are inconsistent results regarding whether positive effects of sleep on memory recall last over a longer time (Cordi and Rasch 2021; Rasch and Born 2013). Regarding sleep-mediated memory benefits, a recent review concluded that “findings and meta-analyses demonstrate that this effect is smaller, more task-specific, less robust and less long-lasting than previously assumed” (Cordi and Rasch 2021). On this background, we believe that it is premature to regard the facilitating effects of sleep on memory consolidation as important for brain health, and we do not address this issue further here. Although this does not mean that sleep cannot have positive effects on consolidation of declarative memories, the nature of such effects is yet to be understood. Modern neural reconfiguration theories about the role of sleep in brain plasticity and learning represent a more general approach to the question and attempt to explain important aspects of brain function. The implications for brain health are less clear, however, so we do not discuss this approach.

## Chronic Sleep Deprivation and Brain Health

**Figure 5.**
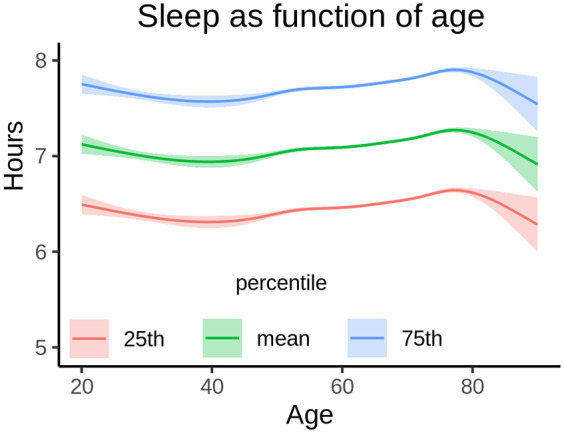
Sleep across the adult life span. Average self-reported sleep duration from 47,029 participants in a study from the Lifebrain consortium. Average reported duration was aproximately 7 hours, with 25% reporting slightly more than 6 hours. Only modest differences were seen across the age span. Shading indicates 95% CI. Adapted from Fjell and others (2023b).

Only half the population sleeps the recommended amount of at least 7 hours per night, and there is a worry that we currently see an “epidemic of sleeplessness” with detrimental consequences for brain health (Hirshkowitz and others 2015; Paruthi and others 2016a, 2016b, 2016c; Watson and others 2015). A study of >1 million participants found that 6.5% consistently sleep <6 hours each night (Kocevska and others 2021), and there is a widely held belief that people in modern societies tend to sleep less than in previous times. A total of 47,000 participants in a study from the Lifebrain consortium (Walhovd and others 2018) reported to sleep on average about 7 hours (Fjell and others 2023b), which is on the lower limit of current expert recommendations (Hirshkowitz and others 2015; Watson and others 2015). As shown in Figure 5, this number is relatively stable across age. Although there is not convincing evidence to suggest that short sleep is more common now than in previous times (Bin and others 2013; Knutson and others 2010; Youngstedt and others 2016) or even in human hunter-gatherers (Yetish and others 2015), it is important to know if chronic short sleep causes lasting negative consequences for brain health.

Abrupt sleep deprivation is associated with acutely poorer neurocognitive performance in many people (Lowe and others 2017), but this approach comes with many caveats (see Fjell and Walhovd 2024 for a discussion), including increased stress and elevated cortisol levels (Siegel 2022). Here, the issue is rather whether short sleep over a prolonged time can have lasting negative effects on brain health, not easily overcome by recovery sleep. It has been suggested that partial sleep loss or insufficient sleep can lead to neural injury and that even shorter periods of sleep deprivation can cause sustained neurobehavioral deficits despite quick normalization of subjective reports of sleepiness and mood with recovery sleep (Zamore and Veasey 2022). For example, one study found that reduction of sleep time by one-third for 10 days in young adults caused neurocognitive changes, some of which normalized after a week of normal sleep while others did not (Ochab and others 2021). Reaction time and subjective sleepiness seem to recover fast even after extended periods of partial sleep deprivation (Axelsson and others 2008; Ochab and others 2021), while accuracy reduction on a 20-minute Stroop task during sleep deprivation was not normalized after a 7-day recovery period (Ochab and others 2021). Two qualifications must be mentioned. First, there are large individual differences, with some participants not showing behavior decrements even with long periods of very short sleep (e.g., 4 hours per Axelsson and others 2008). Second, a few studies in middle-aged and older adults tended to find better tolerance to sleep loss and faster recovery (Duffy and others 2009; Philip and others 2012), which might suggest less sleep need with higher age.

Neural effects of experimentally induced sleep restriction have been studied in rodents (for a review, see Zamore and Veasey 2022), but the implications for the human brain are unclear, as among other things there are differences in neural plasticity in humans vs rodents and in how metabolic waste is cleared from the brain (Cheng and others 2020). Hence, we must attempt to adress the effects of sleep restrictions in human studies. Although observational studies are not well suited to address causal relationships, if short sleep has negative consequences for brain health, we would expect to see traces of this among short sleepers in observational studies also.

**Figure 6.**
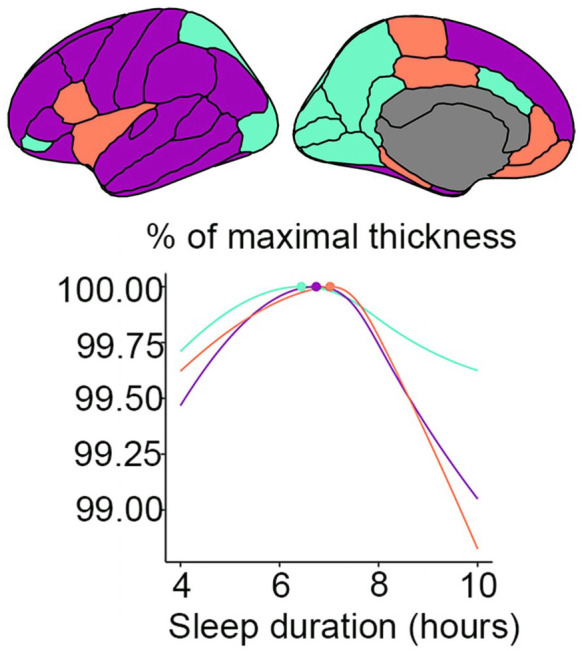
Sleep duration and brain structure. Very short, but especially very long, sleep duration is related to a thinner cortex. Those reporting to sleep 6.5 hours each night had the thickest cortex, the largest regional brain volumes, and the smallest ventricles. The top part of the figure shows three large cortical thickness regions in violet, cyan, and orange, and the corresponding relationship between maximal cortical thickness and sleep duration is depicted below. Adapted from Fjell and others (2023b).

However, in stark contrast to this hypothesis, observational studies converge on ~7 hours of sleep being associated with the highest cognitive function, brain health, and almost any other health outcome. For example, a meta-analysis of 35 studies of sleep duration and mortality found that 7 hours was associated with the lowest risk (Shen and others 2016). Two recent very large studies found 7 hours of sleep to be associated with the highest cognitive performance (Coutrot and others 2022) and lowest dementia risk (Huang and others 2022). Reviewing the literature, we did not find short sleep to be related to poorer brain health as measured by structural MRI in any consistent way. To address the question with good statistical power, we used data from across Europe and the United States as part of the Lifebrain consortium (Walhovd and others 2018). Testing the relationship between self-reported sleep duration and brain morphometry in >51,000 participants across the adult age range, we found that 6.5 hours of sleep was associated with the thickest cortex, largest regional brain volumes, and smallest ventricles (Fjell and others 2023b; Figure 6). Furthermore, longitudinal analyses of almost 4000 participants showed a lack of significant relationships between structural brain changes and reported sleep duration.

In a separate study of the same sample, consistently reporting to sleep <6 hours without feelings of daytime tiredness was not associated with reduced brain health (Fjell and others 2023a). In fact, the 740 short sleepers who reported not experiencing daytime sleepiness or sleep problems/disturbances interfering with falling or staying asleep had significantly larger regional brain volumes than either short sleepers with daytime sleepiness and sleep problems or participants sleeping the recommended 7 to 8 hours. The short sleepers showed slightly lower general cognitive function (0.16 SD), however. Although most of the large-scale studies use self-reported sleep measures, these results were replicated by use of accelerometer-estimated sleep duration, and the associations did not change when controlling for body mass index, depression symptoms, income, and education. A separate longitudinal study of 28,000 participants found faster cognitive decline in individuals sleeping ≤4 or ≥10 hours as compared with a reference group sleeping 7 hours (Ma and others 2020). Between these extreme intervals, sleep duration was not related to different rates of cognitive decline.

Hence, the pattern of results across several large studies does not suggest stable associations between short sleep and reduced brain health when extremely short and long sleep is not considered. The reason for this lack of stable and unidirectional relationships between sleep duration and cognitive function is likely that sleep need differs among individuals and varies over time, interacting with diverse needs and psychological states. This creates a complex relationship between sleep and neurocognitive function, which we need to address to enhance our understanding of the relationship between sleep and brain health. For an in-depth discussion of these issues, see Fjell and Walhovd (2024).

Emphasizing the importance of longer sleep for brain health may have unintended consequences. First, it may create concerns among individuals who, for various reasons, do not obtain the recommended amount of sleep. This aligns with survey findings mentioned in the introduction, where 85% of respondents rated sleep habits as having a “strong” or “very strong” influence on brain health (Budin-Ljosne and others 2022). Such concerns could lead otherwise healthy adults to seek unnecessary medical treatment, including hypnotic drugs. Second, anxiety about inadequate sleep may become self-fulfilling, exacerbating sleep problems. Therefore, we believe that it is essential that communications regarding potential brain health risks associated with short sleep be evidence based, sensitive to effect sizes, and considerate of individual differences in sleep needs and tolerance to shorter and longer sleep durations.

## The Way Forward

Challenges to a better understanding of the role of sleep in brain health centers on unclear causality, mechanisms, and effect sizes. There may be no quick fix to meet these challenges. We believe that it is necessary to consider that changes in brain health often unfold over long timescales and that it consequently can take years or even decades before pathologic changes in the brain, such as upbuilding of amyloid plaques and neurofibrillary tangles, are followed by clinical symptoms. This means that studies need to follow participants over a long time to track the evolution of changes in sleep, cognition, and brain health markers. We propose two research avenues to gain more knowledge.

### Observational Studies: Longitudinal Studies over Extended Time Intervals

Recent progress has yielded new possibilities for collecting this type of data with less effort and cost. Combining sleep registration from wearable devices with repeated app-based subjective sleep measures will allow researchers to collect extensive time series data. Furthermore, blood-based biomarkers of brain health are increasingly accurate, approaching what can be obtained with much more invasive or expensive measures (Barthelemy and others 2024). If such data are coupled to more extensive testing with neuropsychology, brain MRI, other relevant biomarkers, and polysomnography at regular intervals, it will be possible to describe the coupling and timing of changes more accurately. Although still observational, such an approach will allow a more accurate description of the temporal ordering of changes in sleep vs brain health markers.

### Experimental Studies: Experiments Involving Gradual Changes and Longer Follow-up Times

Experiments represent the gold standard for investigating causality but often suffer from low ecological validity. For example, the average sleep reduction across 71 experiments on sleep and cognition was 3.83 hours, which is ~50% of baseline sleep (Lowe and others 2017). Such abrupt and large reductions, usually lasting for only a couple of days, do not resemble habitual variations in sleep duration and likely have no effect on brain health in the long run. The effects of short-term sleep deprivation paradigms cannot easily be extended to real-life “chronic sleep deprivation,” and it is therefore difficult to assess the effects of sleep on brain health using such designs. In contrast, a scheme of gradual changes in sleep patterns happening over longer time spans with frequent brain health testing may provide insights more readily applicable to real-life situations. As mentioned previously, low-threshold information can repeatedly be collected through wearable devices, app-based data collections, and low-cost brain health biomarkers. This approach also allows focusing on the substantial individual differences in responses to sleep changes (Tucker and others 2007), which are crucial to understand.

Both these avenues are demanding and require substantial efforts on the part of the researcher and the participants. Nevertheless, it is our opinion that sleep research needs to heighten the bar to move the field forward in more fundamental ways.
